# Lessons Learned From Over 20 Years of Telemedicine Services in India: Scoping Review of Telemedicine Services Initiated From 2000 to 2023

**DOI:** 10.2196/63984

**Published:** 2025-10-15

**Authors:** Osama Ummer, Anjora Sarangi, Arjun Khanna, Diwakar Mohan, Kerry Scott, Amnesty LeFevre

**Affiliations:** 1 School of Public Health, University of Cape Town Cape Town South Africa; 2 Johns Hopkins India Pvt Limited New Delhi India; 3 2X Digital New Delhi India; 4 Department of International Health, Johns Hopkins Bloomberg School of Public Health Baltimore, MD United States; 5 School of Global Health, York University Toronto, ON Canada

**Keywords:** telemedicine, telehealth, teleconsultation, remote consultation, virtual care, eHealth, digital health, scoping review, India

## Abstract

**Background:**

India is home to some of the world’s earliest and largest telemedicine services. Since the first telemedicine services emerged in the 1990s, the growing digitization of health care services has highlighted the potential for telemedicine services to increase access to timely and appropriate care seeking, corresponding to improved health outcomes and cost savings to the individual and health system. Despite this potential, little is known about the varied typologies of telemedicine services providing in India, their design and model characteristics, scale of implementation, and the available evidence on their impact.

**Objective:**

This scoping review aims to identify the characteristics of telemedicine services in India, including the type of telemedicine model, details on the timing of delivery, health services provided, and service delivery channel. Additional details are extracted on the scale of implementation, software used, and evidence gathered, including impact on care seeking, health outcomes, and cost.

**Methods:**

Telemedicine services in India were identified through searches of Google, the Google Play Store, 3 major scientific databases (Embase, PubMed, and Scopus), and a reference review of identified peer-reviewed articles. Included services were restricted to those implemented in India between January 1, 2010, and July 4, 2023, which included humans, and were published in the English language. Once identified, articles were imported to Covidence, and the process of abstract screening was initiated using 2 independent reviewers and a third person to resolve conflicts. Full-text articles were screened, and data were extracted into Microsoft Excel.

**Results:**

A total of 2368 articles were identified, 151 of which were included for the full-text review and data extraction. From the 151 studies, a total of 115 unique services were identified and further classified based on a scale—moderate to large (n=89) and small (n=26). Among moderate- to large-scale services (n=89), 75 used specialized software and 14 used nonspecialized software, such as WhatsApp. On average, 3 new telemedicine services were initiated annually from 2000 to 2019, and the growth of new services occurred predominantly in the private sector. Evidence was available for 43% (32/75) of the telemedicine services. While 21 services reported on some facet of the quality of care, no studies systematically assessed quality of care. Where structured surveys were reported, questions were often leading, used longer Likert scale response options, and asked respondents about broad constructs subject to varied interpretations (eg, quality of care or satisfaction). Additional details on model characteristics, reach, and impact are presented.

**Conclusions:**

The widespread proliferation of telemedicine services in India has much potential to improve access to and continuity of timely and appropriate care seeking for health. However, improved evidence demonstrating the impact of telemedicine services on care seeking, quality of care, cost, and health outcomes is needed.

## Introduction

In the wake of the COVID-19 pandemic, there has been increasing demand for the remote delivery of health care services using information communication technologies (ICTs), including mobile phones, tablets, and computers [1-3]. Telehealth is defined as the use of ICTs to support and promote remote clinical health services, health education, public health, and health administration [4,5]. Telemedicine is a subset of telehealth that focuses on the use of ICTs for the “provision of health care services, including the exchange of medical information, diagnosis, treatment, and monitoring of patients who are not physically present with the health care provider” [5].

The World Health Organization (WHO) classifies telemedicine services into one of two model types: (1) patient-to-provider*,* where telemedicine services are conducted between patients seeking health care services and health care providers, or (2) provider-to-provider, where telemedicine is conducted between 2 or more health care providers to provide specialized input or second opinions [5]. Telemedicine services may be delivered in real time (synchronously), where live interactive sessions are involved, or in a deferred mode (asynchronously), where data are stored and information is sent remotely through a remote client or patient monitoring, also known as telemonitoring. The main channels for providing telemedicine services include audio calls, SMS text messages, email, audio-video calls, smartphone or customized applications, and picture archive and communication systems [5].

India (population of 1.4 billion) is home to some of the world’s earliest and largest telemedicine services [6]. Emerging first in the 1990s, early telemedicine services were designed and implemented by the Indian Space Research Organization (ISRO), using satellite communication to connect providers in frontline health facilities (“spokes” or peripheral hospitals) with specialists in tertiary hospitals (“hubs”) to deliver health care service remotely [7]. At the turn of the century, the ISRO expanded its partnership to include the Apollo private hospital network, a partnership that has evolved to include premier public sector facilities, including the All India Institute of Medical Science (AIIMS) New Delhi, the Postgraduate Institute of Medical Education and Research (PGIMER) Chandigarh, and the Sanjay Gandhi Postgraduate Institute of Medical Sciences, and additional private hospitals (Apollo, Aravind Eye Care, and Narayana Hrudayalaya) [7-9]. By 2015, the ISRO network had grown to include over 245 hospitals (205 district and rural hospitals and 40 superspecialty hospitals) across India [9].

In the wake of COVID-19, additional telemedicine services have continued to emerge. Most notably, eSanjeevani, a national telemedicine service, was launched by the Government of India in early 2019. eSanjeevani includes both patient-to-provider and provider-to-provider telemedicine services and is currently operational in 31 states and union territories across India. Since September 2023, with the support of nearly 200,000 registered providers, eSanjeevani is reported to have served over 162 million patients through 1.08 million health and wellness centers (spokes) and 14,007 secondary or tertiary hospitals (hubs) [10].

The growing digitization of health care services in India and elsewhere globally has highlighted the potential for telemedicine services to increase access to timely and appropriate care seeking, corresponding to improved health outcomes and cost savings to the individual and health system. Despite this potential, little is known about the varied typologies of telemedicine services providing in India, their design and model characteristics, scale of implementation, and the available evidence on their impact. Improved understanding of the services implemented to date, particularly at scale in India, may help to guide the efforts of future telemedicine services in other low- and middle-income countries where the disease burden is highest and the need for improved access to timely and appropriate health services is greatest.

This scoping review aims to describe the characteristics of large-scale telemedicine services initiated between 2000 and 2023 in India and to present an overview of the evidence available on these services. Study findings are anticipated to improve understanding of the vast expanse of telemedicine services offered in India and provide insights into the design, implementation, and available evidence on the impact of these telemedicine services.

## Methods

### Overview

This review adopted a scoping review methodology to map the breadth of telemedicine initiatives in India and generate insights into their design, implementation, and reported impact. In keeping with the objectives of scoping reviews, no formal assessment of risk of bias or methodological quality was undertaken [11]. The review was conducted in accordance with the framework proposed by Arksey and O’Malley [12] and reported following the PRISMA-ScR (Preferred Reporting Items for Systematic Reviews and Meta-Analyses extension for Scoping Reviews) guidelines, as given in Multimedia Appendix 1.

### Search Strategy

A comprehensive and multisource search strategy was used to identify telemedicine services in India. The primary information sources were the 3 major scientific databases, such as Embase, PubMed, and Scopus. Additionally, a Google web search was conducted to identify gray literature and programmatic reports, and the Google Play Store was searched to capture relevant mobile health applications. The reference lists of included articles were also reviewed to identify additional eligible studies. This combined approach ensured that both published evidence and real-world implementations not indexed in traditional databases were included. Detailed search strategies for each source are provided in Multimedia Appendix 2.

### Eligibility Criteria

Telemedicine was defined as including (1) a health care expert (doctor, nurse, physical therapist, or nutritionist) who makes (2) decisions tailored to a specific patient profile, through (3) a digital solution, including phone, computer, or tablet. “Formal” telemedicine services were defined as digital communication sanctioned by the organization and used according to a protocol. Telemedicine services were further categorized based on the reported scale of their implementation and considered to be moderate to large in scale if they met one or more of the following criteria: (1) a minimum of 1000 app downloads or patients reached, catered to, or consultations conducted, and (2) implemented in >1 hospital or geographical location.

Included telemedicine services were restricted to those that included humans, were published in the English language between January 1, 2010, and July 4, 2023, and pertain to services in India. Studies were excluded if they (1) were 1-way direct-to-beneficiary applications that provide information only, (2) relied on informal technology use by providers, such as personal telephone calls or patient contact solely on publicly available chat applications (eg, WhatsApp), (3) focused on data capture, workflow support applications, clinical decision-making algorithms, or job aids, including those that use artificial intelligence to render a diagnosis or are used by providers to screen patients in the course of home visits, (4) pertained to e-training or e-mentoring services, or (5) self-monitoring services, including those involving artificial intelligence or chatbots. Articles focusing solely on the technical specification of internet connectivity, book reviews, and conference proceedings were also excluded.

### Study Selection and Data Charting

Once identified, articles were imported to Covidence (Veritas Health Innovation Ltd), and the process of abstract screening was initiated using 2 independent reviewers and a third person to resolve conflicts. Full-text articles were screened by 2 independent reviewers and a third person to resolve conflicts. Data from the full-text articles were extracted into Microsoft Excel. To ensure alignment across reviewers with the data extraction, weekly meetings were held across the study team. Senior investigators additionally conducted spot checks of articles to review their classification and the data extracted.

### Data Items

**Figure 1:**
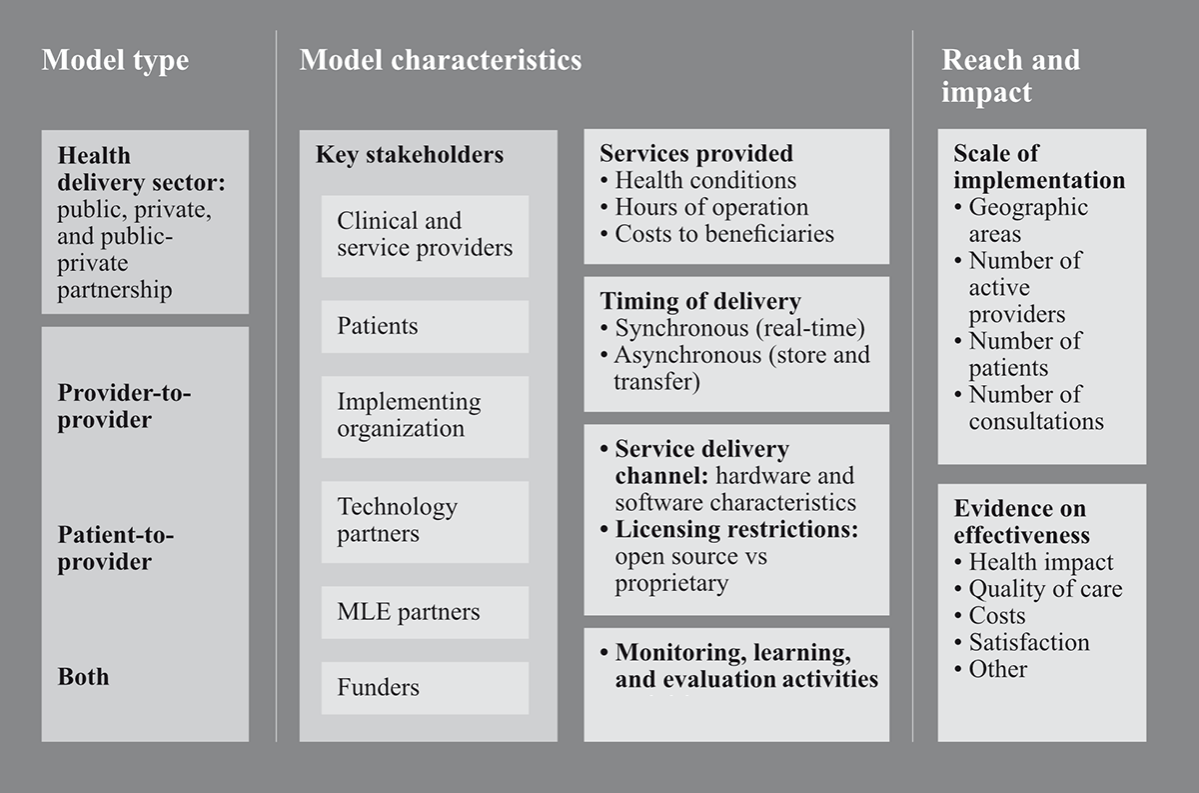
Extraction domains used for assessing telemedicine model type, model characteristics, and reach and impact. MLE: monitoring, learning, and evaluation.

Figure 1 summarizes the extraction domains across three broad categories: (1) model type, (2) model characteristics, and (3) reach and impact. The model type includes the health delivery sector (public, private, or public-private partnership [PPP]) and the WHO classification type (provider-to-provider, patient-to-provider, or both). Model characteristics include key stakeholders, services provided, timing of delivery, service delivery channel, licensing provisions, monitoring, and learning and evaluation activities. Reach and impact include details on the scale of implementation and evidence on effectiveness where reported.

### Critical Appraisal of Evidence

Given that this is a scoping review and not a systematic evidence synthesis or meta-analysis, we did not assess the quality of evidence reported in individual articles. Rather, the goal of this scoping review was to identify the full range of telemedicine services, including those for which peer-reviewed articles have not been published. Findings from peer-reviewed articles on the effectiveness of telemedicine sought to provide a broad overview of the landscape of evidence across disparate types of research and areas of inquiry.

### Synthesis of Results

Efforts to synthesize details on the model characteristics of the telemedicine service sought to follow the framework in Figure 1. Efforts to collate evidence on effectiveness drew from the evaluation categories depicted in WHO’s guidance on the monitoring and evaluation of digital health interventions [13].

## Results

### Overview

**Figure 2:**
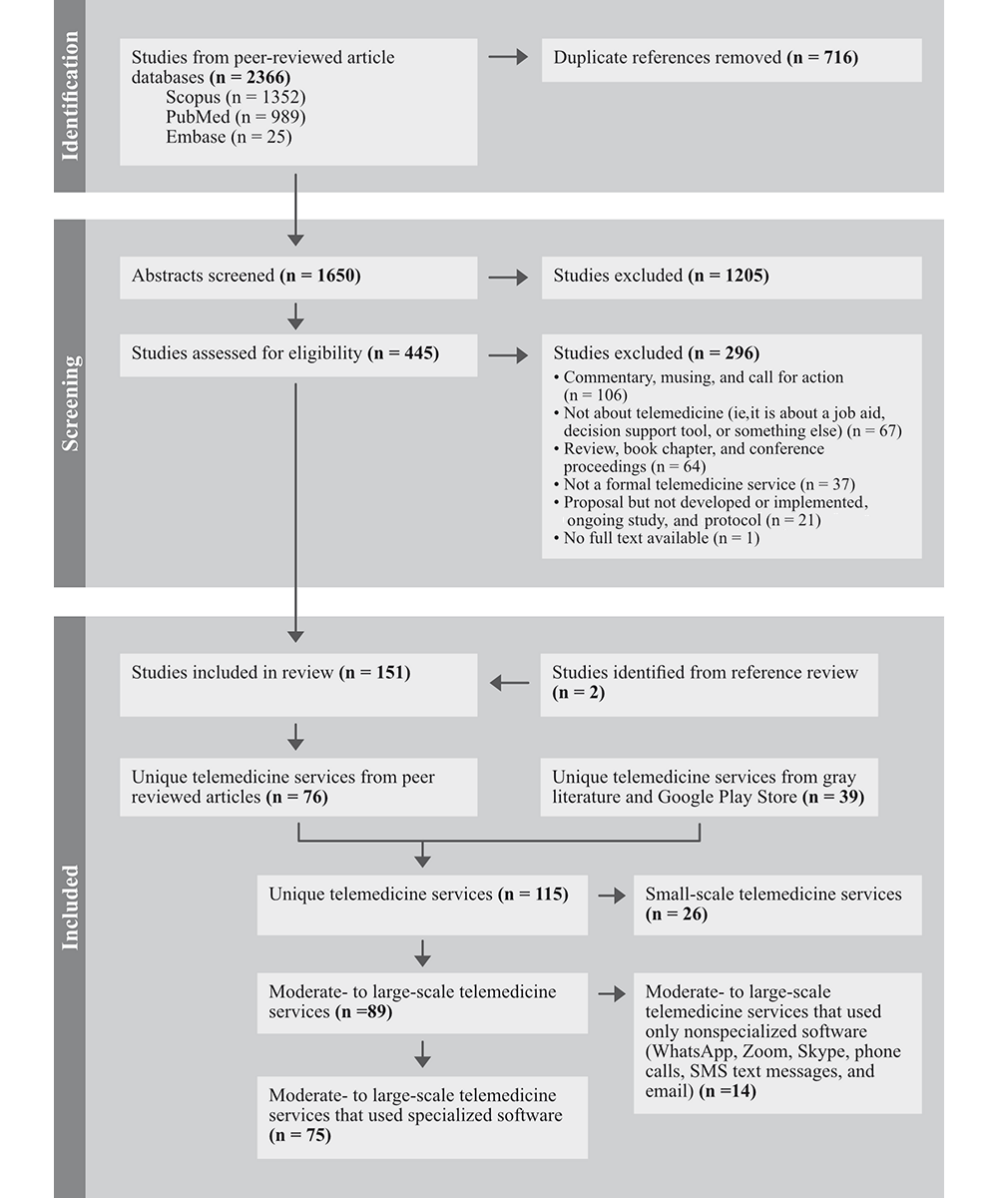
PRISMA (Preferred Reporting Items for Systematic Reviews and Meta-Analyses) flow diagram.

The PRISMA (Preferred Reporting Items for Systematic Reviews and Meta-Analyses) flow diagram in Figure 2 provides a summary of the screening process. From the peer-reviewed article databases, 2366 articles were identified, and after the exclusion of 716 duplicates, the abstracts from 1650 articles were screened for eligibility. Of these, 1205 were excluded, and 445 articles were deemed eligible for full-text review. A total of 151 studies were included for the full-text review and data extraction, including 2 articles identified from the references of other articles.

To identify unique telemedicine services, we categorized peer-reviewed articles by name and additionally reviewed the gray literature and Google Play Store. A total of 115 unique telemedicine services were identified (76 from the peer-reviewed literature and 39 from gray literature and the Google Play Store). Unique telemedicine services were further classified based on (1) scale and (2) reported use of specialized software. Among the 115 unique services, 89 (77%) were classified as being moderate to large in scale, and 26 (23%) were small. Large scale is operationalized as those services that met one or more of the following criteria: (1) a minimum of 1000 downloads, patients, or consultations, and (2) implemented in >1 hospital or geographical location. Among the 89 moderate- to large-scale services, 75 used specialized software and 14 used nonspecialized software, such as WhatsApp. The tables and figures that follow present extracted data for the unique moderate- to large-scale services that reported using specialized software (n=75).

### Characteristics of Telemedicine Services

**Figure 3:**
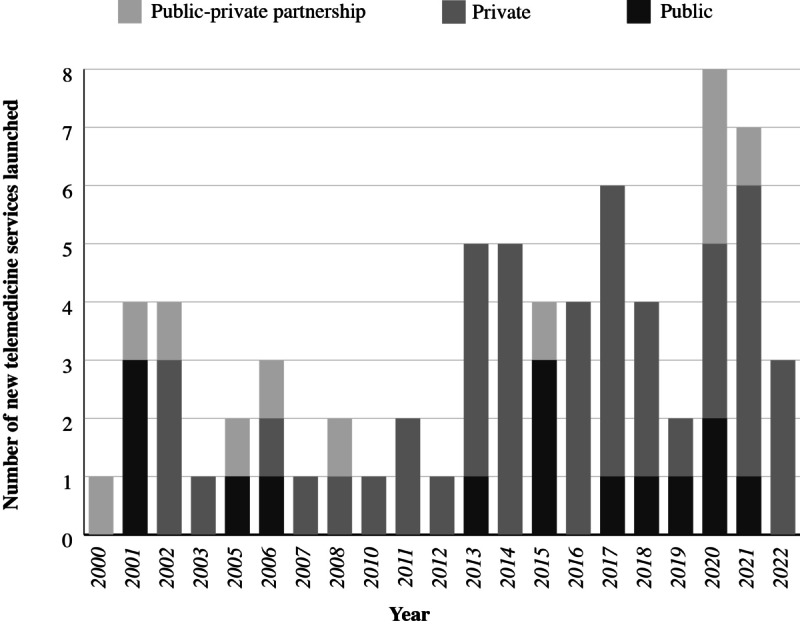
Number of moderate- to large-scale unique services using specialized software initiated over time.

Figure 3 shows the distribution of moderate- to large-scale unique telemedicine services using specialized software initiated or launched between 2000 and 2023. On average, 3 new telemedicine services were initiated annually from 2000 to 2019, and the growth of new services occurred predominantly in the private sector. The start of the COVID-19 pandemic in 2020 corresponds to an increase in the number of new telemedicine services.

### Model Characteristics

Table 1 presents summary characteristics of moderate- to large-scale telemedicine services using specialized software in India. Out of 75 services, 64% (48/75) were delivered by the private sector, while 19% (14/75) were public sector and 17% (13/75) were PPP. Nearly half (37/75) of the services were provided through a patient-to-provider model, 24% (18/75) provider-to-provider, and one-third (20/75) using both patient-to-provider and provider-to-provider models. Services were provided in real time (synchronous) for 69% (52/75), while 28% (21/75) of services delivered both synchronous and asynchronous services, and 3% (2/75) delivered only asynchronously. While most services (52/75, 69%) offered multispecialty care covering 2 or more health domains or conditions, one-third (23/75) focused on condition-specific care (eg, ophthalmology or mental health). All services in both public (14/75, 19%) and PPP (13/75, 17%) sectors were provided with limited (nominal charges for outpatient registration) to no fees. In the case of private sector services (n=48), service fees ranged from US $2.40 (INR 200) to US $7.21 (INR 600) per service, and for some services, monthly subscription fees ranging from US $18 to US $32 (INR 1500-3000) were charged depending upon the services beneficiaries subscribed to. For some private sector telemedicine services, beneficiary charges occurred indirectly through the purchasing of insurance and other employee wellness schemes.

**Table 1:** Characteristics of moderate- to large-scale telemedicine services using specialized software in India (n=75).

Telemedicine service characteristics			Values, n (%)
**Health delivery sector**			
	Public	14 (19)	
	Private	48 (64)	
	Public-private partnership	13 (17)	
**Model type per WHO^a^ classification**			
	Provider-to-provider	18 (24)	
	Patient-to-provider	37 (49)	
	Both	20 (27)	
**Timing of delivery**			
	Synchronous or real-time	52 (69)	
	Asynchronous	2 (3)	
	Both	21 (28)	
**Health domain or condition**			
	Multispecialty	52 (69)	
	Condition specific (eg, ophthalmology or mental health)	23 (31)	

^a^WHO: World Health Organization.

### Key Stakeholders

Table 2 outlines the details of key stakeholders engaged in the implementation of moderate- to large-scale telemedicine services. The earliest telemedicine services involving the public sector were initiated by the ISRO with support of other government bodies, including the Department of Information Technology, Ministry of External Affairs, Ministry of Health and Family Welfare, and the state governments [14]. More recent telemedicine services have been led by the Ministry of Health and Family Welfare at the national level, in coordination with state governments for implementation (14/75, 19%). The public sector included both models of service delivery, that is, patient-to-provider (5/14) and provider-to-provider (2/14). In contrast, the majority of private sector services (48/75, 64%) were patient-to-provider (30/48), through one of two categories: (1) networks of hospitals (16/48, 33%) or (2) technology service providers (32/48, 67%) who created technology solutions. The latter included business-to-business for third-party health care providers (n=8) and business-to-consumer technology solutions for patients and providers (n=24).

**Table 2:** Key stakeholders of moderate- to large-scale unique telemedicine services using specialized software (n=75).

Health delivery sector		Public (n=14), n (%)	PPP^a^ (n=13), n (%)	Private (n=48), n (%)	
**Model type**					
	Provider-to-provider	2 (14)	5 (38)	11 (23)	
	Patient-to-provider	5 (36)	2 (16)	30 (62)	
	Both	7 (50)	6 (46)	7 (15)	
**Implementing organization**					
	Networks of hospitals	N/A^b^	N/A	16 (33)	
	Technology service providers (B2B^c^)	N/A	N/A	8 (17)	
	Technology service providers (B2C^d^)	N/A	N/A	24 (50)	
**Clinical and service providers**					
	MBBS doctors or higher-level specialists	14 (100)	13 (100)	48 (100)	
	Dentists	0 (0)	0 (0)	11 (23)	
	AYUSH^e^ practitioner	1(7)	1 (8)	7 (15)	
	Allied health services	4 (29)	4 (31)	11 (23)	
**Patients (age group)**					
	All age groups	13 (93)	13 (100)	48 (100)	
	Specific (pediatric)	1 (7)	0 (0)	0 (0)	

^a^PPP: public-private partnership.

^b^N/A: not applicable.

^c^B2B: business to business.

^d^B2C: business to consumer.

^e^AYUSH: Ayurveda, Yoga and Naturopathy, Unani, Siddha and Homeopathy.

All 75 (100%) telemedicine services had MBBS doctors or higher-level specialists as clinical providers, while 25% (19/75) included access to allied health services, 13% (10/75) to dentists, and 12% (9/75) to AYUSH (Ayurveda, Yoga and Naturopathy, Unani, Siddha, and Homeopathy—the 6 Indian systems of medicine) practitioners. Among beneficiaries, only 1 telemedicine limited services to pediatric patients, while the remainder (74/75, 99%) catered to patients of all age groups. Technology, monitoring and evaluation, and funding of the services were either not reported or limited. Detailed description of key stakeholders for each telemedicine services is provided in Multimedia appendix 3.

### Scale of Implementation

The scale of implementation for the moderate- to large-scale telemedicine services using specialized software is summarized in Multimedia Appendix 4. Services reported their scale of implementation using a wide range of parameters, and no common standard has yet been developed. Thus, we gather information on scale across the following parameters based on available information: (1) geographic areas (state and districts) of implementation, (2) number of registered providers, (3) number of spokes and hubs, (4) number of patients served or treated, (5) number of consultations (overall or daily) or prescriptions, and (6) number of downloads on the Google Play Store. Information for at least 1 scale parameter was reported in 75 telemedicine services.

Among public sector services, as of July 19, 2023, eSanjeevani reported the largest number of registered providers (n=185,100) and health facilities (>100,000 primary health clinics and >13,000 secondary and tertiary hospitals) and is operational across 31 states and union territories across India. The total number of patients served was reported to exceed 138 million, and over 10 million consultations were carried out from November 2019 to July 2023. Among PPP models identified, Apollo telehealth services reported providing services in over 350,000 telemedicine centers, Apollo clinics, and common service centers, and 73 Apollo hospitals across 14 states in India. From 2000 to 2023, Apollo services reportedly reached over 13 million patients and delivered over 16 million teleconsultations. Among private sector–only models (n=48), 17% (8/48) reported having conducted over 1 million consultations. Practo, a private sector service that launched in 2008, provides services through over 0.1 million doctor partners. Textbox 1 provides a brief overview of the 5 largest telemedicine services in India that use specialized software.

**Overview of the 6 largest telemedicine services in India that use specialized software.**

**eSanjeevani**

- Public
- Largest
- Model type: patient-to-provider and provider-to-provider, along with assisted telemedicine service
- Provides chat and audio-video consultations, real-time and asynchronous telemedicine, free of cost, with state service doctors, Ayushman Bharat Health Account integration, a multilingual interface, and health services covering allopathic care and Ayurveda—with variation across states, where some also include Homeopathy. Available as mobile and web-based application and facility-based online system. Implemented as hub-and-spoke model, where hubs are either secondary or tertiary care centers (community health centers, district hospitals, or medical college hospitals), dedicated telemedicine centers, or primary health centers, and spokes are Ayushman Bharat health and wellness centers.

**Indian Space Research Organization (ISRO)**

- Public-private partnership (PPP)
- First telemedicine network
- Used satellite communication
- Model type: provider-to-provider, along with assisted telemedicine service
- Provides audio-video consultations, real-time telemedicine, free of cost, state service and private specialist doctors, and allopathic health services. Available through facility-based online system. Implemented as hub-and-spoke model, where hubs are specialty hospitals (government and corporate) and spokes are remote, rural, or district hospitals or telemedicine mobile units.

**National Telemedicine Network**

- Public
- First fiber-optic–based telemedicine network
- Model type: provider-to-provider
- Provides audio-video consultations, real-time telemedicine, free of cost, state service doctors, and allopathic services. Available through facility-based online system. Implemented as a tiered hierarchy of support, wherein primary health centers and community health centers were upgraded with broadband to provide telemedicine services, district hospitals provide telemedicine support to these community-level facilities, and super specialty hospitals (All India Institute of Medical Science) and medical colleges provide a further tier of support.

**Apollo group of hospitals**

- Private and PPP
- First PPP-based telemedicine provider
- subsidiary of the largest hospital network
- Model type: patient-to-provider and provider-to-provider, along with assisted telemedicine service
- Provides audio-video consultations, real-time telemedicine, state service doctors in PPP, private specialist doctors, and allopathic services. Available as mobile and web-based application and facility-based online system. Implemented as hub-and-spoke model, where hubs are superspecialty Apollo hospitals, including Apollo Chennai and Apollo Hyderabad, and spokes are (1) Apollo clinics and Apollo telemedicine centers (private model) and (2) mostly government health centers (PPP model). Patient-facilitated subset of common service centers serves as access points created under the National e-Governance project of the Government of India. Other facilities include ordering medicines over the internet through Apollo pharmacies and patient bookings over the internet through the Ask Apollo application across India.

**Aravind Eye Care (teleophthalmology)**

- PPP
- Model type: provider, along with assisted telemedicine service
- Launched as a PPP model in partnership with ISRO as a mobile eye unit. Later established a private sector model as the Aravind teleophthalmology network. Network of eye hospitals with primary care vision centers, as well as secondary and tertiary specialty centers. An example of a disease-specific telemedicine service.
- Provides audio-video consultations, real-time and asynchronous telemedicine, private specialists, and allopathic services. Implemented as hub-and-spoke model, where hubs are Aravind eye hospitals, including Madurai and Chennai, and spokes are (1) vision centers across Coimbatore, Tirunelveli, and Madurai in Tamil Nadu; (2) community outreach via mobile eye care units and eye camps; and (3) selected diabetic centers across Tamil Nadu for diabetic retinopathy screening.

**Practo**

- Private
- Launched as a platform for booking doctor appointments, which evolved to include a telemedicine application
- Largest online directory of doctors
- Model type: patient-to-provider
- Provides audio-video consultations, real-time telemedicine, cost per service, allopathic and AYUSH (Ayurveda, Yoga and Naturopathy, Unani, Siddha, and Homeopathy) health services, and private doctors enrolled in Practo working in various clinics or hospitals across selected cities in India. Available as a mobile and web-based application. Other facilities include a hospital and clinic management system that is compliant with Ayushman Bharat, a patient management application, and the ability to order medicine and laboratory tests online. The platform is ISO 27001 certified, and its data centers are Health Insurance Portability and Accountability Act (HIPAA) compliant.

Over a quarter (21/75, 28%) of the moderate- to large-scale services that used specialized software were being implemented in 1 state. The remaining services are implemented in multiple states—17% (13/75) in fewer than 10 states and 12% (9/75) in 10 or more states—or did not report any geographical location (4/75, 5%). All telemedicine service applications identified from the Google Play Store (28/75, 37%) were accessible in all states across India. However, for some of these (7/28, 25%), accessibility within states was limited to either major cities or certain parts of the state.

Beyond the distribution of telemedicine services across and within states, information on the number of “registered” or “active” providers was reported for only 20% (15/75) of services. For those services that reported this information, the number of active or registered providers ranged from 5 to 0.5 million, with 33% (5/15) reporting 100,000 or more providers. Telemedicine reach in terms of the number of patients served, treated, or “lives saved,” or the number of consultations or prescriptions provided, was reported for 61% (46/75) of services. For the 46 services that reported reach, 36 (78%) served less than 1 million patients or provided consultations, 8 services had between 10 and 20 million, and only 2 services reported more than >100 million patients served (eSanjeevani and Practo). Among the telemedicine applications in the Google Play Store, 61% (17/28) had fewer than 1 million downloads, and 39% (11/28) had more than 1 million, ranging up to >100 million downloads (4 telemedicine services).

### Evidence on Effectiveness

We examined peer-reviewed research articles for evidence on the effectiveness of the 75 moderate- to large-scale telemedicine services. We considered an article to include evidence of effectiveness if it provided information on processes, outcomes, or impact. This included but was not limited to studies on reach, quality of care, economic evaluation, or provider or patient perceptions of the service. Details on the evaluation were extracted, including study design, methods, and findings (Table 3).

**Table 3:** Summary of evidence on effectiveness.

Evidence of effectiveness					Moderate- to- large-scale telemedicine services reporting effectiveness (n=75), n (%)			Articles reporting on effectiveness (n=84), n (%)
**Inputs**								
	Technological readiness			20 (27)			34 (40)	
	Patient readiness			6 (8)			9 (11)	
	Provider readiness			15 (20)			28 (33)	
	Structural readiness			14 (19)			17 (20)	
**Processes**								
	Technical care			14 (19)			20 (24)	
	Interpersonal and respectful care			11 (15)			21 (25)	
	Technological performance			9 (12)			11 (13)	
	Patient-provider engagement with technology			5 (7)			8 (10)	
**Outcomes**								
	Experience of care			17 (23)			36 (43)	
	Costs, time savings			14 (19)			25 (30)	
	Health outcomes			24 (32)			52 (62)	
	Provider capacity (at the spoke level)			3 (4)			5 (6)	
	Equity			3 (4)			4 (5)	
	Gender inclusion			2 (3)			2 (2)	
**Economic evaluation**								
	Cost-effectiveness or cost-utility			6 (8)			7 (8)	
	Cost outcome (telemedicine service costing analysis)			4 (5)			4 (5)	
**Data sources**								
	System-generated data analysis			7 (9)			8 (10)	
	Structured survey (patients and providers)			19 (25)			39 (46)	
	Qualitative methods: in-depth interviews and focus group discussions			6 (8)			6 (7)	
	Medical record review			23 (31)			45 (54)	
	Clinical observation			3 (4)			3 (4)	
	Vignettes			1 (1)			1 (1)	
**Study design**								
	**Descriptive**							
		Surveillance	0 (0)			0 (0)		
		Ecological correlation	0 (0)			0 (0)		
		Cross-sectional (prevalence)	22 (29)			50 (60)		
		Case report	6 (8)			6 (7)		
		Qualitative	6 (8)			6 (7)		
	**Analytic**							
		Experimental with randomization	0 (0)			0 (0)		
		Quasi-experimental	3 (7)			8 (11)		
		Observational: cohort	6 (8)			7 (7)		
		Observational: cross-sectional	8 (12)			10 (13)		
		Observational: case-control	1 (1)			1 (1)		

Evidence on effectiveness was available for 43% (32/75) of the services, reported across 84 articles. PGIMER Chandigarh’s telemedicine service was the most studied in terms of service effectiveness, with 10 articles published. The National Institute of Mental Health and Neuro Sciences Bengaluru telemedicine service was a close second with 7 articles, while 14 of the 32 services with evidence on effectiveness had just 1 published article covering this topic (Table 3). See Multimedia Appendix 4 for details by telemedicine service and article.

### Evidence on Effectiveness Related to Health Outcomes From Analytic Studies

Given the large number of studies across a range of designs, we focus here on a synthesis of findings from the analytic research on health outcomes. Evidence of the effectiveness of telemedicine on health outcomes (which includes impact on patient access to care, diagnosis, and morbidity) was reported in 52 of 84 articles for 24 of 75 telemedicine service (Table 3), of which 18 articles for 11 services provided analytic evidence (see Multimedia Appendix 5). The remaining articles reported solely descriptive findings.

Within the set of 18 analytic articles on health outcomes, none were randomized controlled trials. A total of 7 were quasi-experimental studies on 3 services: the Pediatric HIV Telemedicine Initiative in Maharashtra [15-17], the World Health Partners’ Sky Program in Uttar Pradesh and Bihar [18-20], and Aravind Eye in Tamil Nadu [21]. The clinical management of children living with HIV in centers linked with the Pediatric HIV Telemedicine Initiative was better compared to nonlinked centers [15-17,22]. Fewer patients were lost to follow-up at the centers with the Pediatric HIV Telemedicine Initiative, but there was no difference in the proportion of patients with delayed treatment once the telemedicine service reached its later phase of implementation [15]. The World Health Partners’ Sky Program showed no improvement in the quality and coverage of maternal health services at the population level [20], no improvement in treatment for childhood diarrhea and pneumonia, nor reduced prevalence of these diseases before and after implementation [18], nor did it change provider knowledge [19]. Opening an Aravind Eye telemedicine center staffed by mid-level (nonphysician) providers led to a significant increase in overall network visit rates and rates of eyeglasses prescriptions for the population living within 10 km of the new center [21].

The 11 remaining analytical studies on health outcomes consisted of 5 observational cohort studies on 5 services [23-28], 5 observational cross-sectional studies on 5 services [29-33], and 1 observational case-control study [34]. Among the observational cohort studies, 2 found that telemedicine was associated with patient improvements; patient mental health scores significantly improved post telepsychiatry treatment in Goa [23], and patients showed a significant reduction in hemoglobin A_1c_ (HbA_1c_) test result from baseline to follow-up while receiving telemedicine support through the Diabetes Tele Management System at Jothydev’s Diabetes and Research Center in Kerala [25]. One found no significant difference in functional assessment of “overdentures” (dentures anchored to teeth or modified roots) fabricated by newly graduated students who were guided remotely through provider-to-provider telemedicine versus guided in person at a university teaching hospital [26]. Furthermore, 2 reported that telescreening for retinopathy of prematurity was suitable to assess incidence over time [24,27].

The observational cross-sectional studies found that the use of telemedicine for diagnosis was equal to in-person models or brought added benefit. The 2 found comparable levels of diagnosis between telemedicine and in-person care: school hearing tests conducted by doctors through a remote audiometer, Distortion Product Otoacoustic Emissions system, and video-otoscope compared to doctors in person [29], and diabetic retinopathy screening conducted by doctors through Dr Mohan’s Diabetes Specialties Center’s teleophthalmology compared to doctors in person [31]. Comparing the diagnosis of head and neck tumors made in person by clinicians at Amrita Institute of Medical Sciences, Kochi, versus remotely by colleagues in the United States found high concurrence, low differential diagnosis, and some additional diagnoses [32]. Sankara Nethralaya’s telescreening model diagnosed a higher prevalence of diabetic retinopathy compared to the in-person ophthalmologist-based screening camp model and found more sight-threatening retinopathies [33]. Finally, a higher portion of children went for diagnosis referral to telediagnostic auditory brainstem response (ABR) compared to in-person ABR (97% taken to telediagnostic ABR appointment vs 80% taken to ABR appointment) [30].

The observational case-control study compared virtual diabetes care using the Diahome app to hospital outpatient service use and found that app users had a greater reduction in HbA_1c_ (but higher triglycerides throughout) [34]. The remaining studies on health outcomes were descriptive, describing patient outcomes without a comparator.

### Evidence on Costs and Cost-Effectiveness

Data on patient or provider costs for telemedicine services were reported in 21 studies. The predominant means of measuring costs was through structured surveys, which asked respondents about perceived savings of time and money [35-39], future willingness to pay for teleconsultation costs [40], or actual costs incurred. Regarding the latter, in a limited number of studies, a broad range of cost-related outcomes were assessed, including distance traveled to seek care [21,41-47], food and overnight charges [41], consultation and clinical costs [20,21,41,44,46-50], waiting time [42], and reported lost workdays [44-47]. These were used to collectively estimate costs and cost savings attributed to telemedicine services from a range of perspectives.

Costing analyses, which presented data on the costs of a single telemedicine service, were reported in 4 articles. These studies sought to present evidence on the telemedicine costs needed to establish the service [18,20,41]. Data on the cost-effectiveness and cost-utility of telemedicine services were reported in 8 articles for 9 moderate- to large-scale services. The methods, including the perspective from which costs and effects were derived, the primary and secondary data sources, the analytic time horizon used, and sensitivity analyses conducted, varied widely across studies, which impeded efforts to draw cross-cutting syntheses of findings.

## Discussion

### Overview

Scoping review findings led to the identification of 2368 articles from which 151 studies and 115 unique telemedicine services were identified and further categorized based on their scale of implementation and use of specialized software. Among moderate- to large-scale services (n=89), 75 used specialized software in isolation or augmented with telephone calls, WhatsApp, Zoom, and other nonspecialized software. Of these 75 services, 64% (48/75) were in the private sector, and the rest were either public or in partnership with private actors. The patient-to-provider model was the model that nearly half (37/75) of the telemedicine used to deliver their services. Telemedicine services were provided in real time (synchronous) for 69% (52/75), and 28% (21/75) delivered both synchronous and asynchronous services. Evidence was available for 43% (32/75) of the services.

Efforts to differentiate telemedicine services based on their scale of implementation and use of software sought to narrow emphasis in a crowded space, removing the “‘noise” of services established ad hoc within a limited geography or health setting, or without the software arguably needed to scale or accommodate the structural and procedural access controls for handling sensitive personal health data. Use of nonspecialized software may stem from user preferences, wherein providers and patients are more comfortable using existing software already on their phones, or may be driven by specialized software shortcomings. In situations where the specialized software crashes or has limited functions (ie, is only suitable for booking appointments or cannot be used for bidirectional sharing of photos and documents), patients and providers may shift to nonspecialized software. This ongoing use of nonspecialized software has enabled telemedicine services to scale but may have some drawbacks. Using specialized software allows each consultation to be integrated with electronic medical records, enabling backend data on call duration and other parameters to be tracked. In cases where the use of nonspecialized software persists, facilities may want to ensure that providers use telemedicine only on official phones, thereby protecting patient data and ensuring separation of work and personal life for providers.

Data on the typologies of telemedicine models sought to distinguish between provider-to-provider, patient-to-patient, and hybrid models. The fact that public sector services used both models suggests that telemedicine is being operationalized as a health system–strengthening intervention in addition to improving patient access to services by the government. By comparison, in the private sector, the implementation of telemedicine services seemed to focus on the use of telemedicine to expand accessibility and reach.

We found that many departments in large hospitals such as AIIMS New Delhi, Jawaharlal Institute of Postgraduate Medical Education and Research, PGIMER Chandigarh, and Apollo used the hospital-wide telemedicine services in different ways, according to their department’s needs. For instance, at AIIMS New Delhi, we found that 6 departments were using telemedicine and that some had used it for over 6000 patients (eg, pediatrics) [51], while others had used it for just 314 (eg, oncology palliative medicine) [52]. Some reported using only special software, while others reported augmenting this software with WhatsApp or telephone calls.

Study findings on the evolution of telemedicine services in India cement India’s place as a global leader in the use of technology for health. In other low-resource settings, the field is characterized by fragmentation and driven by private sector and nongovernment organization–led models with limited scale and reach. Nigeria is home to several telemedicine initiatives, including the World Telehealth Initiative [53], which aims to expand health care access through a clinical mentorship service in Opoji, Nigeria, and Hudibia (established in 2013), which is an application-based solution that allows users to search and see doctors through videoconferencing or to book a face-to-face appointment [54]. In Ghana, a recent review of telemedicine services [55] identified a small number of services, including Bima, which uses a direct-to-patient model to provide health advice and succinct health education to Ghanaians. Elsewhere regionally, HelloDoctor in South Africa [56] and Babyl Rwanda [57] are private sector models that aim to bolster access to medical doctors and nurses as well as a range of clinical and laboratory services directly to the phones of beneficiaries. Data on the uptake of these services are limited.

The wide breadth and variety of telemedicine services, including public and private sector–led and types of telemedicine models (patient-to-provider or provider-to-provider), render comparisons challenging. However, India is unique for a number of reasons. From a supply-side perspective, the government is investing heavily in national telemedicine services via eSanjeevani (established in 2019), which includes both a patient-to-provider and a provider-to-provider model. While there is limited evidence on the reach and impact of both eSanjeevani models, the service has scaled widely [10] with support from Ayushman Bharat Digital Mission and other government initiatives. From a demand side, less than half of women in India report having access to a mobile phone that they themselves can use [58]. Further barriers to women's use of technology [59] are likely to limit the reach and use of patient-to-provider telemedicine services in India, particularly in rural areas. Emerging data on the limited uptake of eSanjeevani’s patient-to-provider model reinforces these challenges.

### Limitations

The large volume of studies has necessitated that we narrow our focus to unique telemedicine services that are moderate- to large-scale and report using specialized software. The central challenge in reporting the scale was that few services publicly list information on the scale of implementation, including the number of active providers and consultations completed. Those that do have listings have varied definitions for key constructs. For example, unique consultations versus the number of patients treated, and active versus registered providers. While we extracted information on the reported evidence generation, given the volume and variety of methodological approaches undertaken, we have not taken into account the quality of evidence reporting.

### Conclusions

The widespread proliferation of telemedicine services in India has much potential to improve access to and continuity of timely and appropriate care seeking for health. However, our findings highlight significant limitations in evidence generation and reporting. Future research is needed to bolster independent evidence gathering on the impact that telemedicine services may have in bolstering equitable access to timely, continuous health services of equivalent or better quality than face-to-face services. Further data on costs to beneficiaries, including any cost savings, as well as assurances that remote service delivery does not compromise beneficiary experiences, are needed.

## Appendix

Multimedia Appendix 1 PRISMA-ScR checklist.

Multimedia Appendix 2 Search strategy for databases.

Multimedia Appendix 3 Key stakeholder characteristics of moderate- to large-scale unique telemedicine services using specialized software.

Multimedia Appendix 4 Table on scale of implementation of moderate- to large-scale unique telemedicine services using specialized software.

Multimedia Appendix 5 Table on evidence of effectiveness of identified unique moderate to large telemedicine services using specialized software.

## Abbreviations

ABR: auditory brainstem response
AIIMS: All India Institute of Medical Sciences
AYUSH: Ayurveda, Yoga and Naturopathy, Unani, Siddha, and Homeopathy
HbA1c: hemoglobin A1c
ICT: information communication technology
ISRO: Indian Space Research Organization
PGIMER: Postgraduate Institute of Medical Education and Research
PPP: public-private partnership
PRISMA: Preferred Reporting Items for Systematic Reviews and Meta-Analyses
PRISMA-ScR: Preferred Reporting Items for Systematic Reviews and Meta-Analyses extension for Scoping Reviews
WHO: World Health Organization
